# Against erasure: Restoring Dr Florence Buchanan's missing image in physiology

**DOI:** 10.1113/EP093649

**Published:** 2026-02-23

**Authors:** Damian M. Bailey

**Affiliations:** ^1^ Neurovascular Research Laboratory, Faculty of Life Sciences and Education University of South Wales Glamorgan UK

## 1 FROM SCIENTIFIC LINEAGE TO HISTORICAL ABSENCE

On 4 December 2024, I was invited by The Physiological Society to contribute to an *In Conversation* session at the annual Member Forum, held at the Royal Society of Medicine in London. Entitled ‘Advancing through Legacy, Building on Breakthrough’, the session brought together – on the same stage and for the first time – all Editors‐in‐Chief from across The Society's family of (five) journals. We were each asked to reflect on a single historic paper from our respective journals: a publication that not only shaped physiology at the time of its appearance, but continues to influence how the field thinks, measures and innovates today.

**FIGURE 1:**
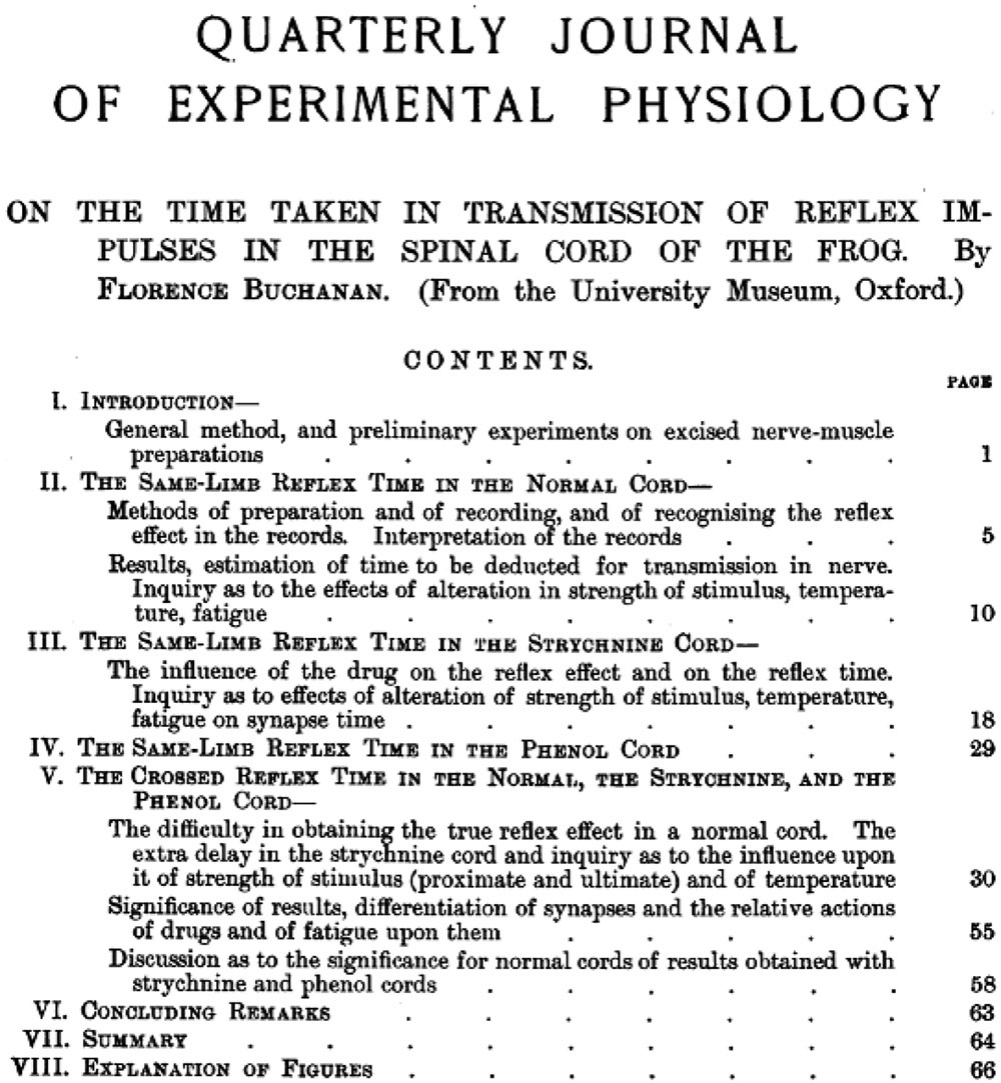
Title page of the 1908 article which was the first paper published in the first issue of the *Quarterly Journal of Experimental Physiology* by Dr Florence Buchanan (Buchanan, 1908) – the journal that would later become *Experimental* Physiology. Reprinted with permission.

My choice was both immediate and clear, shaped not only by the scientific lineage of *Experimental Physiology*, but also by my long‐standing commitment to improving the visibility and representation of women in our discipline (Bailey, 2023). I selected the 1908 paper (Figure 1) of Dr Florence Buchanan (1867–1931) published as the first paper (one of three ‘fearless firsts’, see later) in the first issue of the *Quarterly Journal of Experimental Physiology* (Buchanan, 1908) – the journal that would later become *Experimental Physiology* (Bailey et al., 2023; Bailey et al., 2026). Here, the scientific DNA of our journal was first written.

The extraordinary length of Dr Buchanan's publication (Buchanan, 1908) – nothing less than 67 pages, based on 57 experimental preparations – is itself historically revealing. It reflected the editorial philosophy of Professor Sir Edward Sharpey‐Schafer (1850–1935), who deliberately allowed an author's own voice, argument and experimental detail to unfold with minimal intervention. This permissive approach contrasts sharply with the later, more austere and highly edited style associated with Professor John Newport Langley (1852–1925), whose laconic editorial philosophy and scepticism toward extensive illustration I have discussed previously (Bailey et al., 2023, 2026). Dr Buchanan's paper is also striking for its extensive use of 13 figures, consistent with the Ludwig school's graphical method and its commitment to visualising physiological processes – not as illustrative aids, but as primary data. Rooted in the experimental tradition established by Professor Carl Friedrich Wilhelm Ludwig (1816–1895), this approach transformed physiology into a quantitative science by rendering transient biological events permanently visible. Through such graphical recording (see below), complex neural and muscular phenomena could be measured, compared, and interpreted with a level of precision that fundamentally reshaped experimental physiology. Professor Langley, by contrast, famously regarded figures as superfluous – and, perhaps worse, overly expensive. In Dr Buchanan's work, however, figures are not ornamentation; they are integral to argument, measurement and meaning.

**FIGURE 2:**
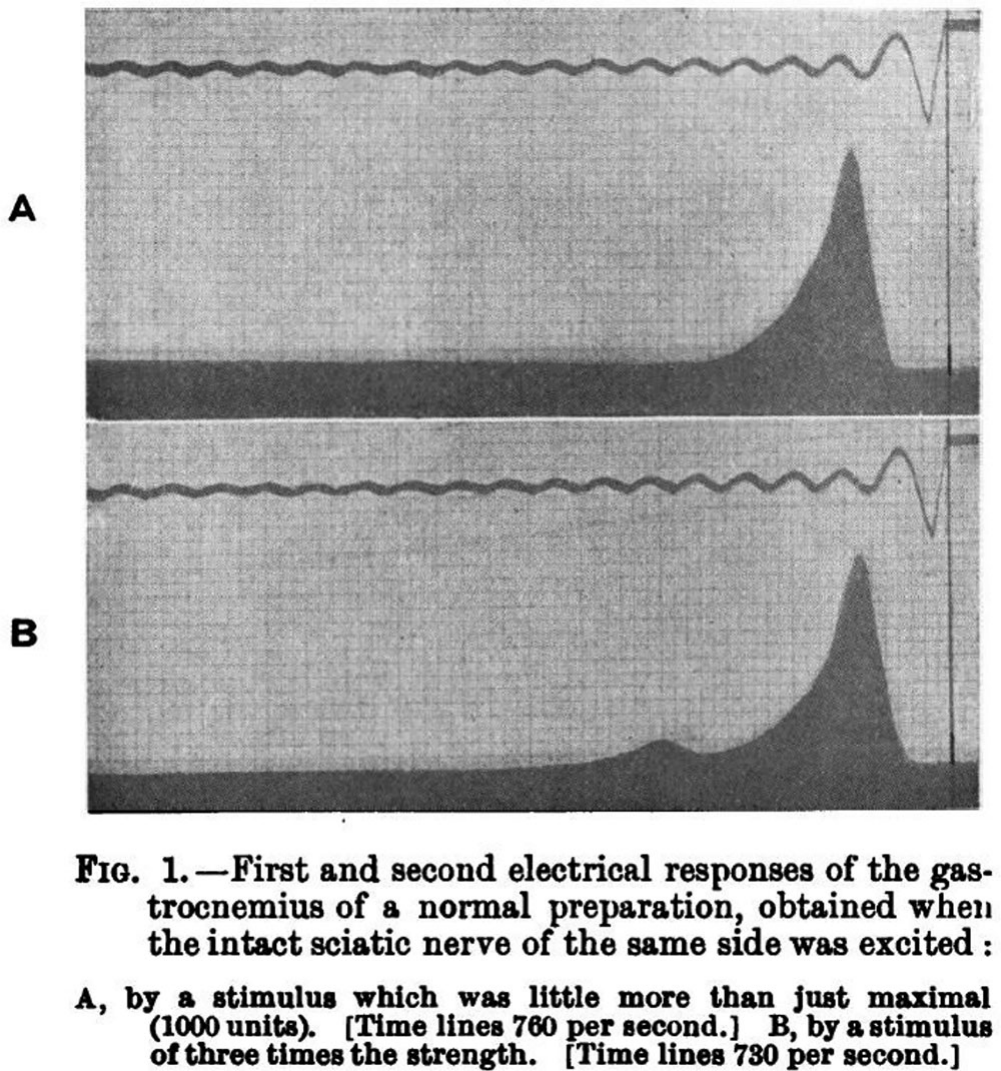
An original recording reproduced from Dr Buchanan's publication (Buchanan, 1908). The paired electrical traces resolve two temporally distinct responses within the same gastrocnemius muscle of the frog following stimulation of the intact sciatic nerve: an initial direct motor response arising from immediate efferent activation, and a second, delayed reflex response generated after afferent transmission through the spinal cord and subsequent return to the muscle. The measurable interval separating these events provides one of the earliest quantitative demonstrations of central synaptic delay. This timing was physiologically profound, as peripheral nerve conduction alone could not account for the observed delay; the remaining interval necessarily reflected synaptic processing within the spinal cord itself. As discussed in the text, this figure – together with the 13 illustrations presented in the paper – exemplifies the Ludwig school's graphical method, in which transient physiological processes are rendered permanently visible and the capillary electrometer trace itself constitutes the primary experimental evidence. Reprinted with permission.

Dr Buchanan's publication (Buchanan, 1908) stands as one of the most rigorous electrophysiological investigations of the early 20th century. At a time when much of physiology relied on blurred mechanical recordings, Dr Buchanan turned instead to electrical measurements using the ‘capillary electrometer’ (note we also lack an original image tied by provenance to Dr Buchanan's bench), resolving events separated by only milliseconds. By eliciting both a direct motor response and a true spinal reflex with a single stimulus and recording both from the same muscle, she isolated what we would now recognise as central synaptic delay (Figure 2). Across dozens of preparations, she reported reflex transmission times of approximately 14–21 ms – values that anticipated later foundational work on synaptic integration and spinal circuitry.

However, what struck me most on rereading this paper in preparation for that *In Conversation* session, was not only its technical elegance, but its intellectual confidence. Dr Buchanan anticipated core principles of modern neurophysiology – synaptic delay, integrative spinal processing, pharmacological modulation of transmission – decades before the conceptual framework to name them formally existed. Her photographic traces remain astonishingly clean, precise, and interpretable even by today's standards. Taken together, this was not merely a ‘fearless first’ paper in a new journal; it was a clear declaration of what quantitative, integrative physiology could – and arguably should – be.

It was only after preparing that presentation – and having read the Blue Plaque Review on Dr Buchanan by Dr Brian C. Clark (Clark, 2025) – that the broader implications of her story came fully into focus. That review distilled, with scholarly clarity, both the scale of her scientific impact and the structural inequities that shaped her career, situating her work within a wider history of exclusion that affected many women in early 20th‐century science. As Dr Clark reflected in that article, ‘…this project, I realised, would not only be about resurrecting her science but reviving, as best I could, the memory of the woman behind it’ (Clark, 2025). My intent here is closely aligned, but more narrowly focused: not to re‐examine Dr Buchanan's science – now so clearly and authoritatively brought to light – but simply to put a face to the scientist whose seminal works have already been restored to their rightful place.

That face belongs to one of physiology's earliest ‘trailblazers’. Dr Buchanan occupies a singular place in the history of The Physiological Society, defined by a series of ‘fearless firsts’ that repeatedly challenged the status quo (Burgess, 2015; Tansey, 2015). She was the first woman to attend a meeting of The Physiological Society, in 1896 – 22 years after its founding – although she did not join the men for dinner, an event that at the time featured live animal experimentation and was regarded as the meeting's highlight. Professor Ernest Starling (1866–1927) famously objected, remarking that ‘it would be improper to dine with ladies smelling of dog – the men smelling of dog that is’ (Evans, 1964). She later became the first (alphabetically) of six women elected to membership of The Society at the AGM in January 1915. This was made possible by a postal ballot courageously proposed by Professors John Scott Haldane (1860–1936) and Langley, culminating in the landmark adoption of Rule 36: ‘Women shall be eligible for membership of The Society and have the same rights, duties and privileges as men.’ This history underscores why representation – particularly women in physiology – continues to matter, a theme actively championed in recent editorials reflecting on other female ‘trailblazers’ including the scientific legacy of Dr Marie Krogh (1874–1943) (Berg, 2024; Cramon et al., 2025).

## 2 ERASURE IN LIFE, MEMORY, AND MEMORIAL

And yet, even with that context established, one absence proved impossible to ignore. Despite her central role in the early history of *Experimental Physiology*, her repeated contributions to The Physiological Society, and her interactions with many of the discipline's most celebrated figures, no photographic or painted likeness of Dr Buchanan is known to exist. Not a portrait, not a sketch, not even a fragmentary archival image. Her contemporaries are visually preserved in abundance; Dr Buchanan, by contrast, survives almost entirely through her data, her prose and the occasional sharp remark recorded in institutional minutes.

This visual absence has, perhaps unsurprisingly, prompted well‐intentioned but problematic attempts at substitution. A photograph recently posted on the History of Oxford/Oxford Explorers Facebook page on 11 October 2025 (https://www.facebook.com/groups/425240434576048/posts/2285742365192503/) was accompanied by the statement: ‘No known portrait of Florence Buchanan survives – yet her legacy is everywhere in Oxford's scientific story. The picture is of an anonymous woman from the late 19th century. This is how I imagine her: thoughtful, determined, quietly shaping the path for generations of women in science.’ While evocative, such images are decontextualised: anonymous period photographs, untethered from textual, biographical or archival evidence. It illustrates how readily absence invites projection when no authentic visual record exists to resist it.

This exclusion is even more striking given that Dr Buchanan was born into social and educational privilege – underscoring that structural barriers to visibility and recognition in physiology operated even when class and access might otherwise have conferred protection. Her *Nature* obituary attributes her scientific ability primarily to her male relatives, underscoring the gendered nature of recognition even after her death.

Heredity may have accounted for Dr Buchanan's scientific skill and enthusiasm, for she was a daughter of the late Sir George Buchanan, Chief Medical Officer of the Local Government Board, and a sister of Sir George Seaton Buchanan and Lady Adam Smith, wife of the Principal of the University of Aberdeen (Nature, 1931).

I confess that, despite having written previously – albeit briefly – about Dr Buchanan (Bailey, 2023), I was blissfully unaware of this absence. On first reading Dr Clark's review when originally submitted to *Experimental Physiology* (Clark, 2025), I contacted the publications office to query what I assumed must be a typesetting error in Figure 2 stating that ‘No known image of Florence Buchanan exists’. My assumption was misplaced – and, in retrospect, telling. A formal correction was issued to remove any ambiguity (Clark, 2025).

Absence, it seems, followed Dr Buchanan not only in life and print, but into memory itself. This imbalance is echoed even in her final resting place. As noted by Dr Clark (Clark, 2025), Dr Buchanan is buried close to Professor John Burdon‐Sanderson – a close family friend, early mentor, and advocate – yet while his grave is marked by a large headstone and decorative iron fence, hers lies modestly behind, inscribed simply with her name, degree and dates. The proximity speaks to connection; the disparity speaks just as clearly to asymmetry in recognition.

## 3 ARTIFICIAL INTELLIGENCE AGAINST ERASURE

That absence is not incidental. It reflects a deeper historical pattern in which women's scientific labour was meticulously documented in text yet rarely rendered visible (Ross et al., 2022). Standing on that stage in London, reflecting on Dr Buchanan's science, while being unable to show her face, crystallised a simple but unsettling realisation: if physiology is a fundamentally visual and experimental discipline, then the persistent invisibility of one of its pioneers is a distortion, not a neutral omission: a wrong that I felt a personal urge to try and put right.

Thus, the primary aim of this editorial – emerging directly from that moment – is explicit: to create an evidence‐based, generative artificial intelligence (AI)‐assisted artist's impression of Dr Buchanan, grounded in her published work and experimental context, and justified as a scholarly act rather than imaginative licence. It represents a gentle attempt not to embellish history, but to confront a silence within it – and to ask what it means for our discipline, past and present, when foundational figures remain unseen. Our collective memories of the discipline are shaped not only by publications, citations and concepts, but by faces, laboratories, benches and instruments. When those images exist almost exclusively for men, the historical narrative becomes subtly but profoundly distorted.

The use of AI‐assisted image generation in this context should be understood not as invention, but as integrative synthesis. It enables textual evidence, methodological detail, architectural setting and material culture – long preserved only in written form – to be assembled into a coherent visual artefact (DaCosta, 2025; He et al., 2025). Crucially, these images are not presented as definitive portraits, but as evidence‐informed visual hypotheses.

This interpretive restraint was shaped in part by Dr Buchanan's personal and social context. As the daughter of one Chief Medical Officer and raised within a milieu of public service, discipline, and professional expectation, she was formed by a culture that valued duty, rigor and quiet contribution. That combination of privilege and constraint – access without full acceptance – was treated as a tonal parameter during image generation, influencing posture, bearing, and the deliberately unsentimental character of the reconstructed scenes, rather than informing any facial, hereditary, or physiognomic inference. The resulting images are therefore explicitly provisional, historically constrained, and open to critique and refinement, which I actively welcome.

Importantly, the alternative to reconstruction is not neutrality, but continued absence. To refuse to visualise Dr Buchanan is to accept, and quietly perpetuate, the very erasure that has characterised her legacy and that which Blue Plaque initiative seeks to correct. AI is employed here not to embellish history, but to interrogate it – to ask what is lost when foundational figures remain unseen. Methodologically, the process was disciplined and iterative, not a creative shortcut. The aim was not aesthetic novelty, but historical plausibility – to ensure that what is illustrated could reasonably have existed, even if it was never recorded – governed by constraint rather than imagination and intended to invite scrutiny rather than demand acceptance.

## 4 TWO IMAGES, ONE CORRECTIVE PURPOSE

**FIGURE 3:**
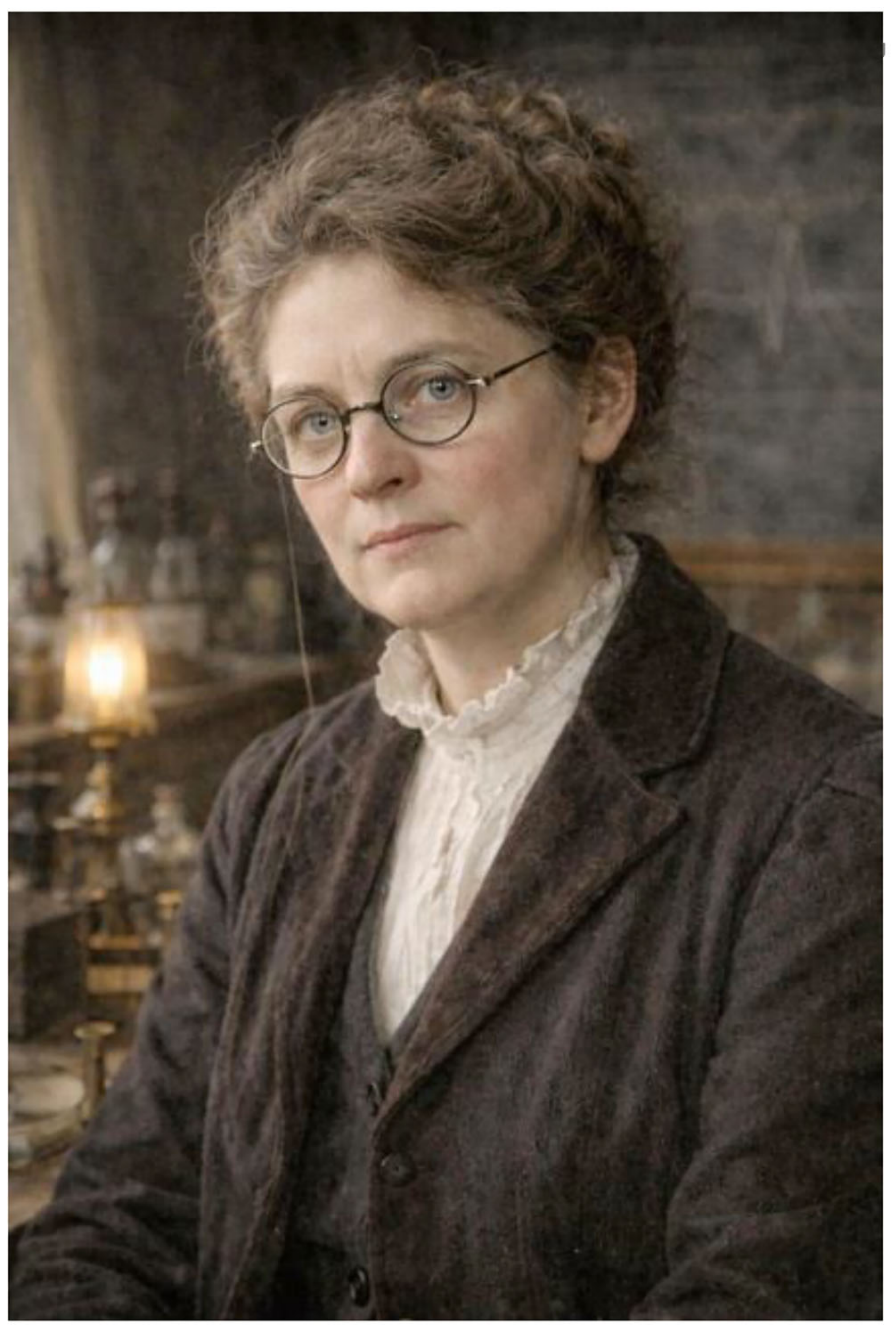
AI‐generated documentary portrait of Dr Florence Buchanan (c. 1907–1909). This image presents an artificial intelligence (AI)‐assisted, evidence‐informed head‐and‐shoulders portrait of Dr Florence Buchanan, created to provide a historically plausible visual presence for a physiologist for whom no known photographs or painted likenesses survive. The portrait was generated through an iterative AI‐based image synthesis process, grounded explicitly in textual information derived from Buchanan's published scientific corpus, contemporaneous descriptions of her working practices, personal recollections by those who knew her, including family accounts that referred to her affectionately as “Aunt Florence” (Mitchison & Squier, 1995), and the material culture of physiology at the turn of the twentieth century. Primary constraints informing the generative model included: (i) temporal anchoring to Dr Buchanan's early forties (c. 1907–1909), corresponding to her period of independent scientific activity following her landmark 1901 *Journal of Physiology* paper (Buchanan, 1901) and contemporaneous with her 1908 contribution to the inaugural volume of *The Quarterly Journal of Experimental Physiology* (Buchanan, 1908); (ii) adoption of early 20th century documentary photographic conventions, including restrained lighting, muted tonal range, and shallow depth of field, consistent with laboratory‐adjacent portraiture rather than formal studio compositions; (iii) historically appropriate Edwardian academic attire consistent with laboratory work rather than formal portraiture; (iv) facial expression and posture informed by the tone and character evident in Dr Buchanan's experimental writing – precise, analytical and unsentimental, and by contemporary descriptions of her as “distinguished, if somewhat difficult”, reflecting a commanding and uncompromising intellectual presence (Mitchison & Squier, 1995); and (v) inclusion of functionally grounded physical attributes supported by biographical and contemporaneous textual evidence, most notably the depiction of thickened corrective spectacles. This choice was guided by the *Nature* obituary's account that ‘for the past ten years she had been handicapped by increasing blindness’ (Nature, 1931) due to a detached retina, suggesting that significant visual impairment was present during the period represented. Accordingly, the spectacles were treated as a practical and historically plausible necessity associated with prolonged visual concentration and fine discrimination required for electrophysiological recording, rather than as an aesthetic or stylistic choice. Additional appearance‐related constraints included a restrained hairstyle, absence of adornment, and a composed, slightly fatigued demeanour, informed by descriptions of Dr Buchanan's intensive laboratory working practices, disciplined economy of her scientific writing, and her fierce independence and resistance to social conformity. These attributes were incorporated as tonal constraints within the AI prompting process, without implying specific or definitive facial morphology. The image was generated using a large‐scale transformer‐based text‐to‐image generative model developed by OpenAI (DALL·E), accessed via the OpenAI Image Generation API. Prompts were constructed exclusively from historically constrained textual descriptors without the use of photographic inputs, visual proxies, or lineage‐based inference. No familial images were available or used in the reconstruction; in particular, no known images of Dr Buchanan's mother, Alice Mary Asmar (n.d.), survive that could have informed facial resemblance. Iterative refinement was employed to identify and remove anachronistic features, idealisation or painterly stylisation. The resulting image should therefore be understood as a visual hypothesis, constrained by documentary and contextual evidence, rather than as a definitive likeness. It is intended to support historical interpretation and scholarly communication, not to imply precise or recoverable facial morphology.

Two complementary images were generated, each serving a distinct scholarly function. The first is a restrained head‐and‐shoulders portrait (Figure 3). Its purpose is intentionally modest: to provide a plausible visual presence for Dr Buchanan herself. It does not seek to dramatise discovery or character, but to address a fundamental asymmetry in the historical record – namely, that we can visualise her male contemporaries with ease, but not her. The image is therefore designed to answer a simple question that history has left unresolved: what might it have looked like to meet Dr Buchanan?

**FIGURE 4:**
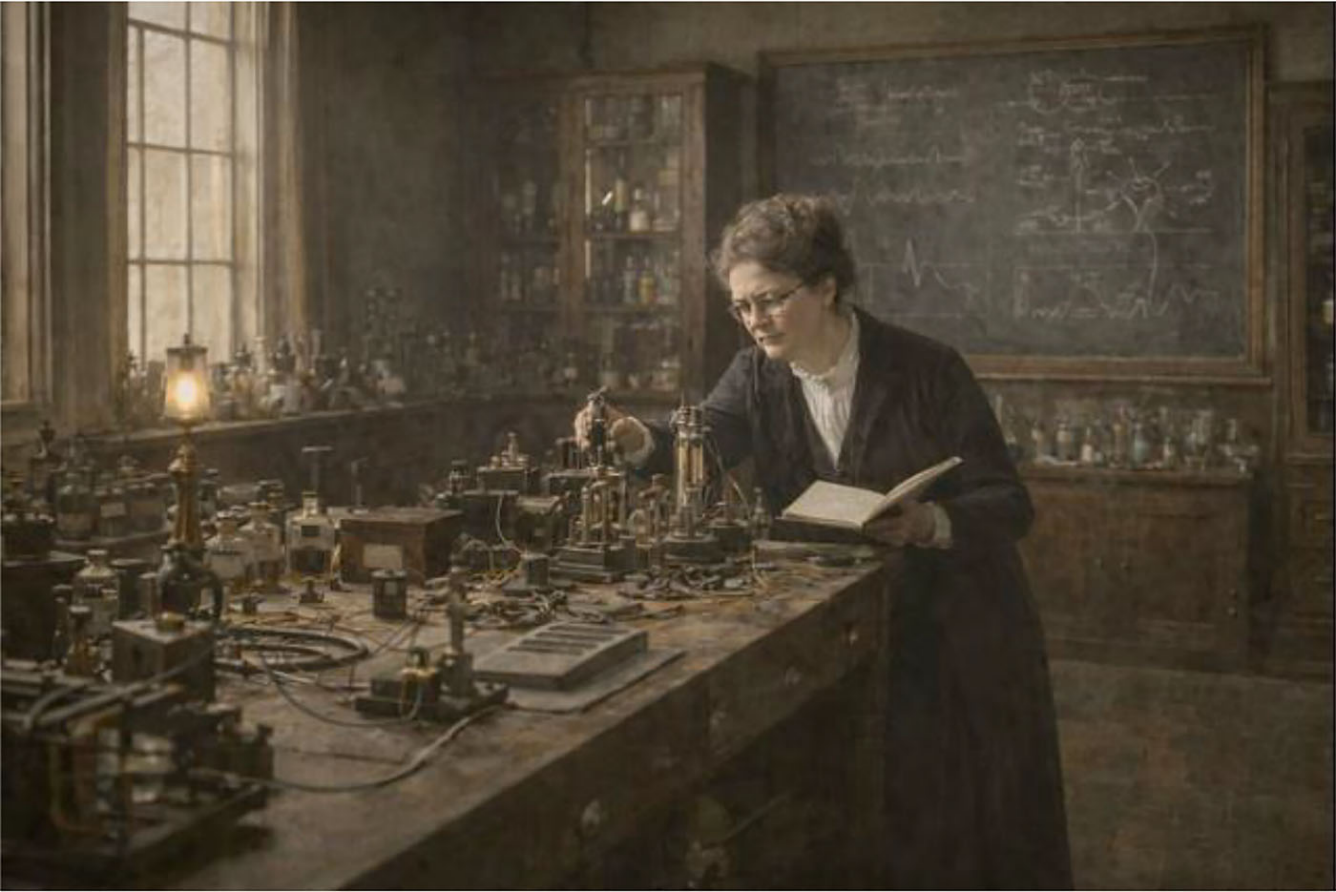
AI‐generated reconstruction of Dr Florence Buchanan at work in an Oxford physiology laboratory (c. 1907–1909). This image presents an AI‐assisted reconstruction of Dr Florence Buchanan situated within the material, technical and intellectual environment of early 20th century experimental physiology. In contrast to Figure 3, which focuses on personal presence, this wider laboratory scene visualises Dr Buchanan as an experimental physiologist at work, embedded within the apparatus, spatial constraints, and methodological practices of her discipline. The reconstruction was derived directly from textual descriptions in Dr Buchanan's published scientific corpus, notably her detailed methodological accounts of electrophysiological investigations of skeletal muscle and cardiac rhythm. These sources informed the selection, configuration and use of laboratory equipment, including capillary electrometers, induction coils, galvanometers, electrodes, mercury‐based glassware and kymographic recording systems. No instruments or technologies beyond those documented in Dr Buchanan's period or experimental practice were introduced. The laboratory architecture reflects late Victorian and Edwardian physiology laboratories associated with Oxford, incorporating heavy wooden benches, dark wood cabinetry, glass reagent bottles, chalkboard notation, and mixed natural and gas or early electric lighting. Dr Buchanan is depicted working alone at the bench, consulting handwritten notes and meticulously adjusting instrumentation. This solitary depiction was intentional, reflecting both the practical realities of experimental physiology and the institutional and social exclusions documented in her career.

The second image places Dr Buchanan within a laboratory setting (Figure 4), shifting the emphasis from presence to practice. Here, she is not the subject of portraiture but the agent of experimental work. This wider scene restores scale, materiality, and context, allowing one to appreciate the physical and intellectual demands of practising experimental physiology during this period. It also reflects the solitary nature of much of her work to which she was entirely devoted and the institutional environment in which she operated. Taken together, the two images are intended to be read in dialogue: one offering human presence, the other professional identity.

Both images deliberately situate Dr Buchanan at approximately 40 years of age (c. 1907–1909) – a period when she was operating as an independent experimental physiologist and contributing at the highest scientific level following (and briefly prior to) publication of her most impactful works (Buchanan, 1889, 1893, 1894, 1899, 1901, 1908, 1909, 1912). This temporal anchoring was critical in that I simply wished to avoid portraying her either as a junior figure under mentorship or as a retrospective icon. Instead, the images place her mid‐career and mid‐experiment, at a point where her scientific authority was already well established, even as she continued to face systemic institutional exclusion.

## 5 WHY REPRESENTATION STILL MATTERS

It would be tempting to treat Dr Buchanan's invisibility as a problem safely confined to the past – that would be a mistake. While the structural barriers faced by women in physiology and the wider life sciences have undoubtedly shifted, under‐representation persists – particularly at senior academic levels and in leadership roles within the discipline (Yfanti, 2025). Women also remain less visible in the historical narratives that define physiology and allied life sciences, including as keynote speakers at major scientific meetings (Larson et al., 2020), as recipients of prestigious prizes and society honours, and as named exemplars of disciplinary ‘greatness’ (Gehmlich & Krause, 2024). Their work is widely cited, yet their presence is less often foregrounded; contributions by women are acknowledged in the scholarly record, but less frequently embodied in the individuals most visibly associated with influence and authority in the life sciences (Ioannidis et al., 2023).

Visual absence plays a subtle but powerful role in this process. When the canonical images of physiology – portraits in lecture halls, society websites, textbooks and journal histories – continue to skew male, they quietly shape expectations about who belongs at the bench, who leads, and whose work is ultimately remembered. In that sense, Dr Buchanan's missing image is not merely a historical curiosity. It is an early manifestation of a problem that remains unresolved. Creating an evidence‐informed visual representation of Dr Buchanan is therefore not only an act of historical recovery, but a contemporary statement: women have always been present at the experimental core of physiology, even when the visual record suggests otherwise.

## 6 LETTING DR FLORENCE BUCHANAN (AND WOMEN IN GENERAL) BE SEEN

If physiology is to take its own history seriously, it must attend not only to what was written, but also to what was never shown. These images represent an attempt – necessarily interpretive, yet rigorously constrained – to address that imbalance. They do not imagine Dr Florence Buchanan into existence; they acknowledge that she was always present and finally allow her to be seen.

Dr Buchanan's absence from photographic history echoes a pattern that remains familiar today. Mothers, who so often serve as custodians of family memory, are frequently missing from the photographs themselves – positioned behind the camera, documenting lives rather than appearing within them. This reflects a broader cultural legacy in which women were conditioned to accept invisibility, to regard modesty and quiet contribution as virtue, and to refrain from seeking recognition. Such absence was not incidental; it was cultural.

While meaningful progress has been made in challenging these ‘norms’, further work remains in collectively insisting that women are not only acknowledged, but visibly present. This editorial seeks to contribute to that effort – to bring women back into the frame, where they have always belonged.

## AUTHOR CONTRIBUTIONS

Sole author.

## CONFLICT OF INTEREST

D.M.B. is Editor‐in‐Chief of *Experimental Physiology*.

## FUNDING INFORMATION

D.M.B. is supported by a Royal Society Wolfson Research Fellowship (Grant No. WM170007).
